# Maize Relay Intercropping with Tobacco Enhances Soil Buffering Capacity and Maintains Yield Under Continuous Cropping

**DOI:** 10.3390/plants15091384

**Published:** 2026-04-30

**Authors:** Qingyao Xu, Xiaopeng Deng, Wengang Duan, Tianyu Li, Yongzhong Li, Jiabo Yang, Jiabin Dong, Yating Liu, Di Liu

**Affiliations:** 1 Tobacco College, Yunnan Agricultural University, Kunming 650201, China; xqy13619436918@163.com (Q.X.); sawengangduan@163.com (W.D.); litianyu88888@163.com (T.L.); liyongzhong168@163.com (Y.L.); 2 Yunnan Academy of Tobacco Agricultural Sciences, Kunming 650032, China; hddxp@163.com; 3 Tengchong Agricultural Technology Extension Center, Tengchong 679100, China; yntcyb@126.com; 4 Zhongke Biotechnology (Yunnan) Co., Ltd., Jianshui 654300, China; 15708775369@163.com

**Keywords:** tobacco, continuous cropping, relay intercropping, microbial community, rhizosphere metabolome, soil enzymes, sustainability

## Abstract

A two-year field experiment was conducted in Tengchong, Yunnan, to evaluate the effects of tobacco monoculture (TM) and maize relay intercropping with tobacco (TIM) on subsequent tobacco growth and the rhizosphere microenvironment. Results showed that TIM significantly increased plant height by 11.8% and maximum leaf length by 12.4% at the vigorous growth stage without reducing yield. Although leaf chloride content increased and the potassium-to-chloride ratio decreased, both remained within high-quality ranges. Relay-cropped silage maize yielded 4.86 t·hm^−2^, adding 1.70 × 10^4^ CNY·hm^−2^. TIM reduced nitrogen accumulation in aboveground tobacco and temporarily lowered soil organic matter and available potassium, while increasing acid phosphatase, peroxidase, and urease activities. Soil bacterial α-diversity increased, with enrichment of beneficial genera, including *Candidatus Solibacter*, *Talaromyces*, and *Penicillium*. Metabolomics identified 1043 metabolites, with upregulation of galactinol, N-acetyl-L-tryptophan, and 3-dehydroshikimic acid, enriched in cyanogenic amino acid and cysteine–methionine pathways. PLS-PM and Mantel analyses indicated that relay-cropped maize indirectly regulates nutrient availability via microbial and metabolic pathways. These results show that maize relay intercropping creates a soil “legacy effect,” shifting the system from direct nutrient competition to microbially mediated nutrient buffering.

## 1. Introduction

In flue-cured tobacco production, crop rotation, intercropping, and relay cropping are widely used to mitigate soil degradation caused by long-term monocropping. These practices improve the physical, chemical, and biological properties of tobacco rhizosphere soils [1]. For example, intercropping tobacco with maize increases soil nitrogen (N), phosphorus (P), and potassium (K) contents [2], and modifies the metabolic profile of the rhizosphere [3]. Relay intercropping is increasingly recognized as a sustainable production system that enhances soil quality, improves nutrient availability, promotes microbial diversity and function, reduces allelopathic effects, and alleviates continuous cropping constraints [4].

Soil microorganisms are key drivers of soil health, nutrient cycling, and crop productivity. By stabilizing and decomposing organic matter (OM), they regulate nutrient dynamics and enzyme activities [5]. Plant species and planting patterns influence soil properties, shape microbial communities [6], and affect processes such as P activation [7]. For example, Yang et al. reported that tobacco–garlic rotation or intercropping increased beneficial rhizosphere microorganisms, including bacteria, actinomycetes, and phosphorus- and potassium-solubilizing bacteria, thereby improving soil quality [8]. Plant species recruit specific microbial assemblages through root exudates and other rhizosphere processes [4]. Accordingly, intercropping optimizes microbial community structure in the tobacco rhizosphere soil and strengthens positive interactions within soil bacterial networks [6,7].

Moreover, intercropping tobacco with sweet potato has been reported to improve soil nutrient availability and microbial diversity by optimizing microbial network structure and enhancing enzyme activity [9]. Accordingly, plant–microorganism interactions have become a central focus in agricultural research [10]. Root exudates represent the primary pathway through which plants regulate the rhizosphere environment, and changes in their composition directly reshape microbial community structure and function [3]. In contrast, continuous cropping often disrupts soil microbial balance and increases the incidence of soil-borne diseases [11]. Well-designed intercropping and rotation systems diversify root exudate profiles and regulate the abundance and composition of soil microorganisms, thereby promoting the recovery of soil ecosystem functions. For example, Lei et al. [3] demonstrated that plant carbon allocation to soil is closely linked to interactions between root exudates and root functional traits. Similarly, Tang et al. showed that intercropping sugarcane with peanuts could enhance fumaric acid secretion from roots, leading to improved soil nutrient status [12]. Do et al. found that root exudates stimulated taxa such as Actinobacteria, Basidiomycota, and Proteobacteria, while suppressing Acidobacteria and Chloroflexi in the rhizosphere [13]. In addition, rotation and intercropping of maize, lily, soybean, or other crops with tobacco could reconstruct soil bacterial community structure and reduce the incidence of tobacco wilt and black shank [11].

In summary, relay intercropping regulates soil microbial communities and metabolic processes through rhizosphere interactions, enhancing nutrient cycling and crop performance. Therefore, this two-year field study aimed to characterize changes in agronomic traits, soil chemical properties, microbial community structure and function, and rhizosphere metabolite profiles under tobacco–maize relay intercropping (TIM). Specifically, we aimed to: (i) determine whether relay intercropping creates a cross-season soil legacy effect that temporally decouples competition from compensation; (ii) reveal how intercropping reshapes microbial co-occurrence networks and metabolite profiles to enhance soil buffering capacity, with emphasis on network modularity and metabolite accumulation patterns; and (iii) assess whether tobacco growth is driven mainly by microbe- and metabolite-mediated indirect pathways rather than direct nutrient supply.

## 2. Results

### 2.1. Effects of Two Consecutive Years of Intercropping Maize on Tobacco

#### 2.1.1. Impact on Agronomic Traits of Tobacco

Compared with monoculture tobacco (TM), tobacco agronomic traits showed no significant differences at the tillering stage after two consecutive years of maize relay intercropping. However, during vigorous growth, plant height and maximum leaf length increased significantly by 11.8% and 12.4%, respectively. At maturity, most traits remained unchanged (Table 1). These results indicate that maize relay intercropping promotes tobacco growth, with the strongest effects during peak growth.

#### 2.1.2. Impact on Nutrient Accumulation in Tobacco

**Figure 1:**
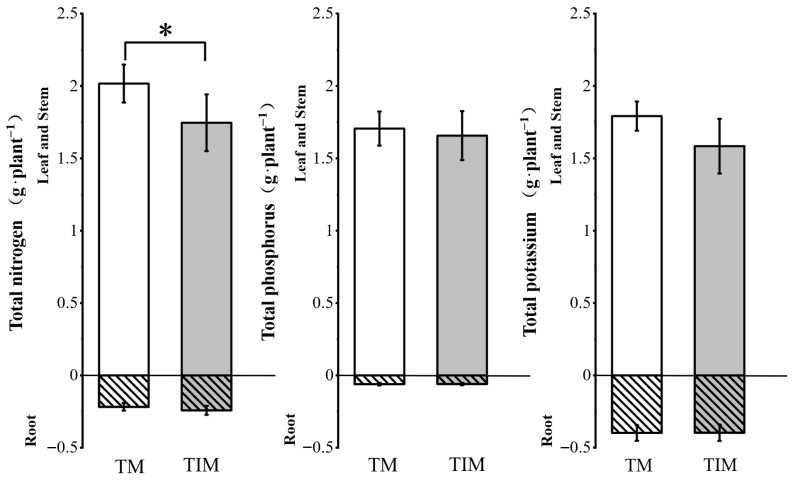
Nutrient status in roots, stems, and leaves of flue-cured tobacco under different cropping systems. Note: TM, continuous tobacco monoculture for two consecutive years; TIM, continuous tobacco intercropped with maize for two consecutive years. An asterisk (*) above bars for the same plant part indicates a significant difference between the two cropping systems at the *p* < 0.05 level.

At maturity, tobacco plants under the TIM treatment showed significantly lower N accumulation in aboveground parts and a declining trend in K accumulation compared with TM (Figure 1), indicating reduced nutrient accumulation.

#### 2.1.3. Impact on the Chemical Composition of Early-Cured Tobacco Leaves

Relative to TM, two-year TIM altered the chemical composition of upper leaves (B2F, Upper Orange II) after curing. Chloride content increased significantly, reducing the potassium-to-chloride ratio. The total nitrogen-to-nicotine ratio and sugar-to-alkaloid ratio also declined by 15.8% and 10.3%, respectively (Table 2). In contrast, middle leaves (C3F, Middle Orange III) showed no significant changes. Despite the decline, the potassium-to-chloride ratio in TIM B2F leaves remained above the recommended threshold for high-quality tobacco (≥6.0), while the decrease in C3F leaves was not significant (*p* > 0.05). The lower sugar-to-alkaloid ratio in B2F may slightly increase leaf irritability, whereas the significantly reduced nitrogen-to-alkaloid ratio suggests more efficient N conversion to nicotine. Overall, maize relay intercropping maintained leaf chemical quality within acceptable limits. Effects were more pronounced in upper leaves but did not compromise industrial usability, while middle leaves were largely unaffected. Under the TIM system, flue-cured tobacco yield and economic return did not differ significantly from TM. However, TIM produced an aboveground biomass of 4.86 × 10^3^ kg/ha, generating an additional profit of 1.70 × 10^4^ CNY/ha (Table 3).

### 2.2. Effects of Two Consecutive Years of Relay Intercropping Maize on Soil

#### 2.2.1. Impact on Soil Nutrients

Compared with TM, TIM significantly increased soil pH, AP, and AN before tobacco transplantation in the following year. Before intercropping, soil pH and AK increased significantly in TIM, whereas OM and AP decreased. At the end of the symbiotic period, soil pH, OM, and AK declined significantly in TIM. After maize maturity following tobacco harvest, OM and AP increased markedly (Table 4).

At the end of the symbiotic period, AK was significantly lower in TIM than in TM (213.8 vs. 233.55 mg·kg^−1^, *p* < 0.05). By maize maturity, AK did not differ significantly between treatments (158.3 vs. 160.4 mg·kg^−1^, *p* > 0.05).

#### 2.2.2. Impact on Soil Enzyme Activity

**Figure 2:**
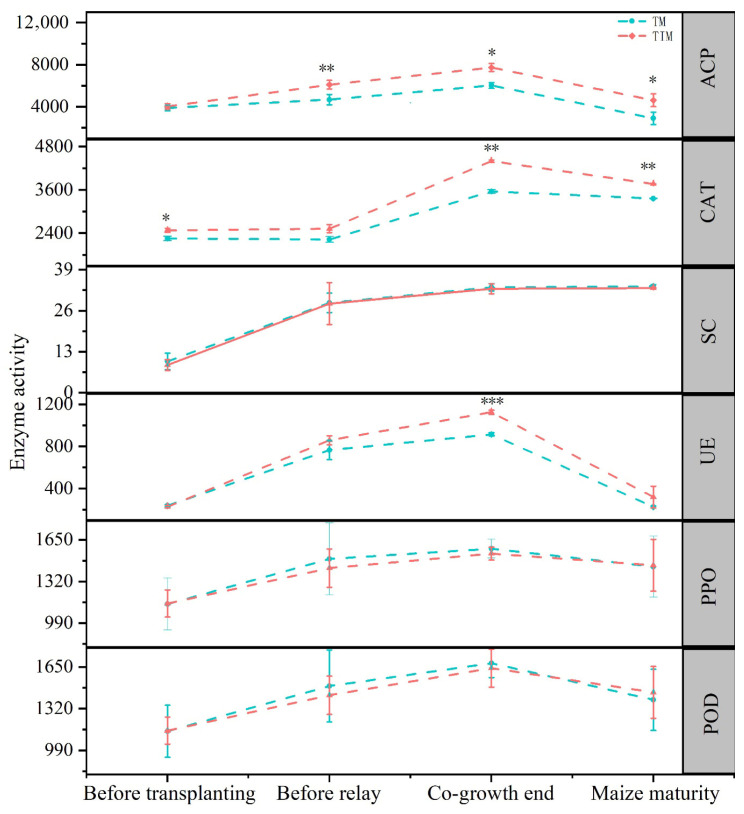
Soil enzyme activities under different cropping patterns. Significant differences are indicated by * for *p* < 0.05, ** for *p* < 0.01, *** for *p* < 0.001. ACP: Acid phosphatase, CAT: catalase, SC: sucrase, UE: urease, PPO: polyphenol oxidase, POD: peroxidase. SC and UE activities are expressed as mg·g^−1^·24 h^−1^; while CAT, PPO, POD, and ACP activities are expressed as nmol·g^−1^·h^−1^. Note: TM and TIM are the same as described in Table 1.

Relative to TM, the TIM system significantly increased soil catalase (CAT) activity before tobacco transplantation in the following year. Before maize intercropping, acid phosphatase (ACP) activity was also significantly increased in TIM. At the end of the symbiotic period, TIM showed notably higher activities of ACP, CAT, and urease (UE). Following the maturation of the subsequent maize crop, both ACP and CAT activities remained significantly higher in TIM compared to TM (Figure 2). Overall, TIM enhanced the activities of key enzymes involved in N and P utilization and OM transformation, thereby promoting continuous nutrient conversion and release within the system.

### 2.3. Effects of Two Consecutive Years of Relay Intercropping Maize on Soil Microbial Diversity and Function in the Crop Rhizosphere

#### 2.3.1. Analysis of Soil Microbial Community α-Diversity and β-Diversity Under Different Planting Systems

**Figure 3:**
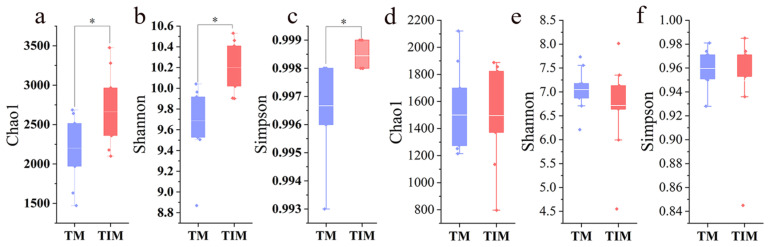
Comparison of Chao index (**a**), Shannon index (**b**), and Simpson index (**c**) for bacteria, and Chao index (**d**), Shannon index (**e**), and Simpson index (**f**) for fungi in the rhizosphere soil of flue-cured tobacco. * indicates *p* < 0.05. Note: TM and TIM are the same as described in Table 1.

After two consecutive years of maize relay intercropping, the TIM treatment significantly increased the Chao, Shannon, and Simpson indices of rhizosphere bacterial communities at tobacco harvest, indicating higher bacterial richness and evenness. In contrast, fungal diversity indices showed no significant differences between treatments (Figure 3).

#### 2.3.2. Analysis of Soil Microbial Communities and Core Taxa Under Different Planting Systems

**Figure 4:**
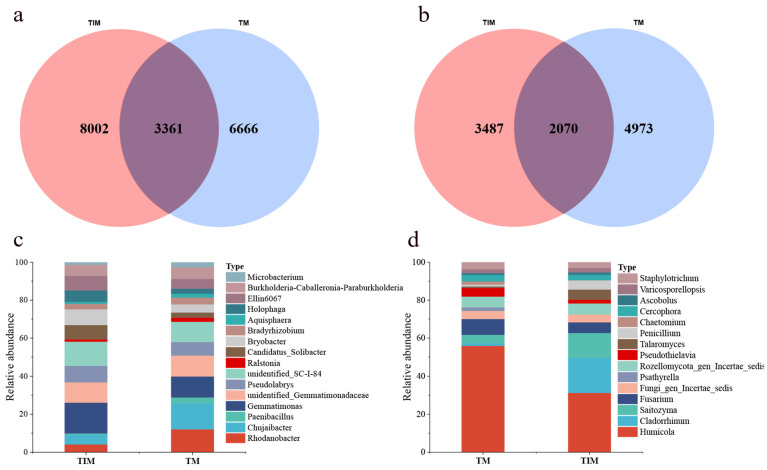
Venn diagram of bacterial (**a**) and fungal (**b**) communities in the rhizosphere soil of flue-cured tobacco under different cropping patterns at the ASV level. Abundance plot of soil bacterial (**c**) and fungal (**d**) community composition. Note: TM and TIM are the same as described in Table 1.

After two consecutive years of maize relay intercropping, rhizosphere analysis at tobacco harvest showed clear microbial shifts. Bacterial amplicon sequence variants (ASVs) were more abundant in TIM (18,029) than in TM (10,530). TIM also produced more unique bacterial ASVs (8002 vs. 6666). In contrast, TIM had fewer unique fungal ASVs than TM (3487 vs. 4973). These results indicate that two consecutive years of maize relay intercropping increased bacterial community uniqueness but reduced fungal community uniqueness in the tobacco rhizosphere (Figure 4a,b).

Analysis of the top 15 genera (Figure 4c,d) showed that the dominant bacterial taxa included *Candidatus Solibacter*, *Chujaibacter*, *Paenibacillus*, and *Pseudolabrys*. Maize relay intercropping increased the relative abundance of *Candidatus Solibacter*, *Paenibacillus*, *Bryobacter* and Ellin6067, while reducing *Rhodanobacter*, *Chujaibacter*, and *Pseudolabrys*. The dominant fungal genera were *Cladorrhinum*, *Talaromyces*, *Penicillium*, and *Psathyrella*. Maize relay intercropping increased the relative abundance of *Cladorrhinum*, *Talaromyces*, and *Penicillium*, whereas it reduced that of *Humicola* and *Fusarium*. Collectively, maize relay intercropping reshaped the rhizosphere microbial community structure of tobacco, selectively enriching beneficial bacterial and fungal genera.

#### 2.3.3. Co-Occurrence Network Analysis of Soil Microbes in the Crop Rhizosphere

**Figure 5:**
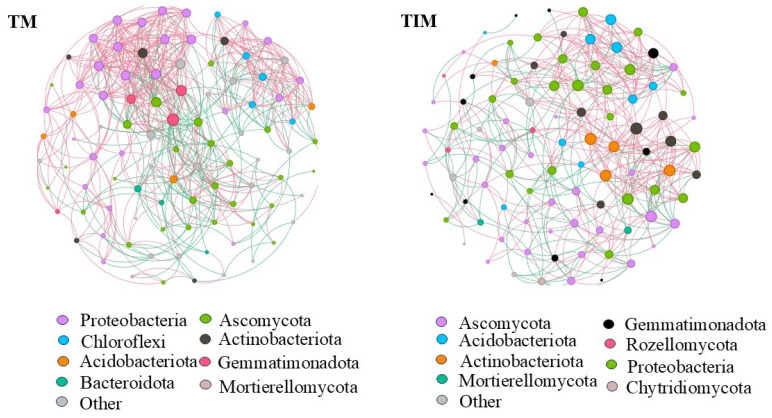
Soil microbial co-occurrence networks under different cropping patterns. Note: Edges represent statistically significantstrong positive (red, Spearman’s ρ > 0.6) or negative (green, Spearman’s ρ < −0.6) correlations. Each node size is proportional to the number of connecting edges (degree). Nodes of the same color belong to the same phylum. The thickness of each connection between two nodes is proportional to the absolute Spearman’s correlation coefficient value (|ρ| > 0.4). Note: TM and TIM are the same as described in Table 1.

Co-occurrence network analysis of the 50 most abundant bacterial and fungal genera under TM and TIM revealed distinct structural differences between the two cropping systems (Figure 5). The TM network was densely interconnected, with higher graph density and more edges. In contrast, the TIM network was more modular, forming loosely connected clusters with fewer edges. In both networks, positive correlations (red edges) exceeded negative correlations (green edges). Compared with TM, TIM showed higher modularity (0.566 vs. 0.519) and average path length (2.966 vs. 2.596), but lower graph density (0.092 vs. 0.123), average degree (8.653 vs. 11.667), total edges (411 vs. 560), and clustering coefficient (0.492 vs. 0.564) (Appendix A Table A1).

#### 2.3.4. Functional Prediction of Soil Microbial Communities in the Crop Rhizosphere

Functional profiles of rhizosphere soil microbial communities were predicted using PICRUSt2 for bacteria and FUNGuild for fungi. Compared with the TM treatment, TIM upregulated bacterial pathways involved in the non-oxidative pentose phosphate pathway and lipopolysaccharide/core oligosaccharide biosynthesis (Appendix A Table A2), while downregulating fatty acid synthesis and β-oxidation. FUNGuild analysis showed that TIM increased the relative abundance of wood-decomposing fungi, particularly *Talaromyces* and *Penicillium* (Appendix A Table A3), and reduced plant pathogenic fungi such as *Fusarium*, along with functions related to mycorrhizal symbiosis. Overall, maize relay intercropping reshaped the rhizosphere functional profile by enhancing bacterial carbon and P metabolism linked to structural biosynthesis, promoting saprophytic fungal activity, and suppressing pathogenic and certain symbiotic groups, thereby improving rhizosphere microecological function.

### 2.4. Effects of Two Consecutive Years of Relay Intercropping Maize on Metabolites in the Crop Rhizosphere Soil

**Figure 6:**
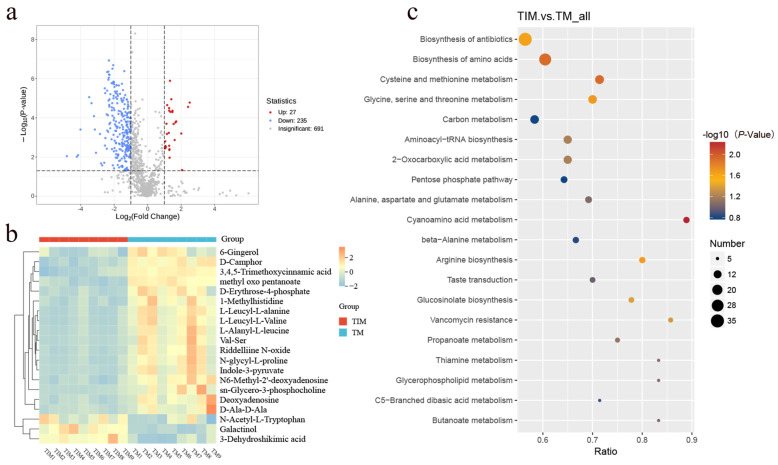
Volcano plot of differential metabolites for the TM vs. TIM comparison (**a**) and cluster heatmap of the top 20 metabolites ranked by VIP scores from the OPLS-DA model (**b**); KEGG enrichment analysis and functional annotation of differential metabolites induced by different cropping patterns (**c**).

A total of 1043 metabolites were detected in the tobacco rhizosphere under the two planting systems. Of these, 262 differed significantly (*p* < 0.05, VIP > 1), including 27 upregulated and 235 downregulated in TIM relative to TM (Figure 6a). Clustering of the top 20 metabolites with the highest VIP scores from the OPLS-DA model showed that, compared with TM, TIM increased metabolites such as N-acetyl-L-tryptophan, galactinol, and 3-dehydroshikimic acid, while decreasing 17 metabolites, including D-camphor and 4-methyl-2-oxopentanoate (Figure 6b). Pathway enrichment analysis indicated that these metabolites were significantly enriched in nine pathways, including cyanoamino acid metabolism, cysteine and methionine metabolism, and amino acid biosynthesis, with most metabolites downregulated (Figure 6c). Collectively, maize relay intercropping markedly altered amino acid and related secondary metabolite accumulation in the tobacco rhizosphere, thereby reshaping metabolic activity and nutrient cycling in the rhizosphere microenvironment.

### 2.5. Correlation Among Soil Factors, Microbes, and Metabolites

**Figure 7:**
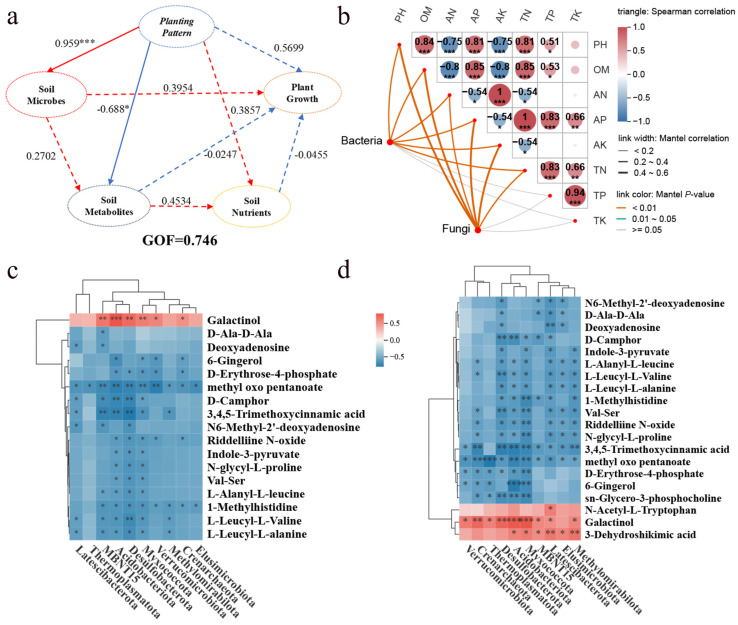
(**a**) Partial Least Squares Path Model (PLS-PM) depicting relationships among soil nutrients, enzyme activities, microbial communities, and metabolites in flue-cured tobacco rhizosphere soil; (**b**) Mantel analysis between soil environmental factors and bacterial/fungal communities; (**c**,**d**) heatmaps showing Spearman correlations between top 20 differential metabolites and bacterial (**c**) or fungal (**d**) phyla. Note: *, **, and *** indicate significant correlations at *p* < 0.05, *p* < 0.01, and *p* < 0.001, respectively (or as defined in the figure).

Partial Least Squares Path Model (PLS-PM) was used to evaluate the effects of maize relay intercropping on tobacco growth and to elucidate the relationships among rhizosphere soil nutrients, microbial abundance, and metabolites (Figure 7a). The model demonstrated an acceptable goodness of fit (GoF = 0.746). The planting system had a strong positive effect on soil metabolites but a significant negative effect on soil nutrients. Furthermore, a Mantel test further indicated that soil environmental factors—including pH, OM, AN, TN, and AK—significantly influenced both bacterial and fungal community composition at the phylum level (Figure 7b). Random Forest analysis identified key bacterial and fungal phyla influencing community structure (Mean_decrease_accuracy > 5), and Spearman correlation analysis between these key phyla and the top 20 differential metabolites (Figure 7c,d) showed that galactinol was positively correlated with multiple bacterial phyla, most strongly with *Acidobacteriota*, whereas most other metabolites showed negative correlations, with the strongest negative association observed between 3,4,5-Trimethoxycinnamic acid and *Desulfobacterota*. Similarly, N-acetyl-L-tryptophan, galactinol, and 3-dehydroshikimic acid were positively correlated with several fungal phyla, whereas other metabolites showed negative correlations. Galactinol showed the strongest positive correlation with *Myxococcota*, while 4-methyl-2-oxopentanoate was most negatively correlated with Thermoplasmatota. Overall, maize relay intercropping drives beneficial restructuring of soil microbial communities and metabolite profiles, thereby enhancing soil fertility. Key metabolites, particularly galactinol and N-acetyl-L-tryptophan, likely function as central signaling molecules mediating microbial community reorganization.

## 3. Discussion

### 3.1. Agronomic Performance and the Soil Legacy Effect

The two-year field experiment showed that TIM significantly enhanced tobacco growth at the vigorous stage (plant height +11.8%, maximum leaf length +12.4%), whereas most traits converged with TM at maturity (Table 1). This “early promotion, late convergence” pattern enables rapid canopy establishment without excessive vegetative growth at maturity—an agronomically desirable trajectory [14]. The vigorous-stage response agrees with the pot study of Ma et al. [15]; however, our field data indicate that it is not driven by concurrent interspecific facilitation, given the minimal co-growth period (maize is relay-interplanted only during lower-leaf harvest). Instead, the effect reflects a cross-season soil legacy established in the first year of relay intercropping [16]. This is supported by higher soil pH, AN, and AP in TIM before second-year transplanting (Table 4). Notably, despite significantly reduced aboveground N accumulation in TIM, plant growth improved during the vigorous stage. Similar temporal trade-offs, including early suppression followed by later compensation, have been reported in potato/faba bean intercropping [17]. Although our relay system differs from concurrent intercropping, a similar mechanism may operate: nutrient competition from the preceding maize reshapes the soil micro-ecosystem, conferring compensatory growth advantages to the subsequent tobacco crop during peak vegetative growth.

Reports on the effects of rotation and intercropping on tobacco leaf quality are inconsistent. Zhang et al. found that a maize pre-crop significantly increased middle-leaf K by 5.80–28.40%, whereas a green manure pre-crop significantly increased upper-leaf total sugar by 11.77–12.27% [18]. Studies from the Xuchang region further show that suitable cropping patterns maintain nicotine at appropriate levels with balanced chemical composition [19]. Here, TIM reduced the K/Cl ratio in upper leaves, but values remained above the high-quality threshold (≥6.0). The total nitrogen-to-nicotine ratio also decreased significantly, indicating more efficient conversion of N into functional alkaloids rather than structural forms. This contrasts with the quality decline typically observed under continuous cropping and suggests that maize relay intercropping sustains productivity while improving nitrogen-use efficiency. Thus, although TIM altered some chemical traits in upper leaves, industrial usability was not compromised [20]. Overall, TIM maintained tobacco yield (1900 vs. 1905 kg·hm^−2^, n.s.) and quality, while generating additional output: silage maize biomass reached 4.86 t·hm^−2^, contributing an extra 1.70 × 10^4^ CNY·hm^−2^ and increasing the land equivalent ratio (LER) to 1.27. This system therefore stabilizes tobacco production while enhancing profitability.

### 3.2. Nutrient Dynamics: Legacy, Competition, and Compensation

Under TIM, N, P, and K showed a consistent three-phase pattern: (i) elevated nutrient baselines before transplanting due to legacy effects; (ii) depletion during co-growth driven by crop competition, accompanied by higher enzyme activities (UE, ACP, CAT); and (iii) recovery or even enhancement after maize harvest.

Higher AN in TIM before transplanting reflects maize straw mineralization from the previous relay season. During co-growth, AN remained similar to TM, but UE activity increased significantly (Figure 2), indicating greater organic N mineralization. However, the released N was rapidly taken up by maize or immobilized by microbes, reducing inorganic N availability for tobacco. This explains the decline in tobacco N accumulation and leaf total nitrogen (TN) [21]. The stable AN pool highlights soil buffering, where N is retained in “stored” forms rather than lost through leaching or volatilization [22].

Elevated AP before transplanting also reflects legacy effects. During co-growth, AP declined to TM levels, while ACP activity remained higher, indicating sustained organic P mineralization. After tobacco harvest, stalk incorporation supplied organic P substrates, and continued ACP activity increased AP beyond TM levels, indicating formation of a “P-activation reserve” [23,24]. The later decline in ACP activity after maize maturity suggests feedback regulation, where sufficient P availability suppresses phosphatase production to conserve energy [25].

K dynamics differed from N and P, being driven mainly by physicochemical processes and residue decomposition. During co-growth, AK was lower in TIM than in TM, while soil pH declined, indicating K mobilization [26]. Organic acids convert mineral K into exchangeable forms, but maize roots rapidly absorb the released K, creating an “activation-uptake coupling” that limits AK accumulation [27]. Residue incorporation is known to increase CAT activity and available K [28]. At maize maturity, tobacco stalk return and sustained CAT activity (Figure 2) promoted K release from decomposing residues, restoring AK to TM levels. Although AK did not exceed TM, leaf K remained unchanged, indicating sufficient K supply.

Collectively, maize relay intercropping enhanced soil nutrient transformation and built nutrient reserves, increasing system buffering capacity—manifested as “competition without collapse” during co-growth and “resilient compensation” thereafter.

### 3.3. Microbial Community Restructuring and Functional Implications

TIM significantly increased bacterial α-diversity (Chao, Shannon, and Simpson; Figure 3), indicating higher richness and evenness, whereas fungal diversity changed little. This pattern is consistent with greater bacterial sensitivity to environmental perturbation and stronger fungal niche conservatism [29,30].

At the genus level, TIM enriched *Candidatus Solibacter* (*Acidobacteriota*), associated with OM-rich soils and potential pathogen suppression [11], consistent with the observed OM accumulation. In contrast, *Rhodanobacter* and *Paenibacillus* declined. *Rhodanobacter* is acidophilic [31]; its reduction aligns with the higher soil pH under TIM. Given its role in nitrification, this decline may slow NH_4_^+^→NO_3_^−^ conversion, favor ammonium retention—the preferred N form for tobacco—and contribute to N buffering [32].

TIM increased saprotrophic fungi, particularly *Talaromyces* and *Penicillium*, and reduced *Humicola* and *Fusarium* (Figure 4d). *Talaromyces* and *Penicillium* are known producers of lignocellulolytic enzymes (e.g., AA3 and AA9 families), which may promote K release from decomposing tobacco residues, consistent with sustained high POD activity at maize maturity [33,34]. Ectomycorrhizal and ericoid mycorrhizal fungi declined. Because mycorrhizal and saprotrophic fungi compete for resources [34,35], reduced mycorrhizae likely freed niche space for saprotrophs. Saprotrophs generally have higher carbon use efficiency; their enrichment suggests more residue-derived carbon is incorporated into microbial biomass and soil OM rather than respired via mycorrhizal networks [36]. This shift from mycorrhizal to saprotrophic dominance provides a microecological basis for OM accumulation and K restitution.

Compared with TM, the TIM co-occurrence network showed higher modularity but lower density and fewer positive edges (Figure 5; Table A1), indicating a shift toward a more modular architecture. Similar patterns have been reported in long-term maize intercropping [37]. Higher modularity is associated with greater resistance to perturbation, as semi-independent modules confine disturbances [38], supporting the observed increase in “buffering capacity.”

PICRUSt2 functional prediction indicated upregulation of the non-oxidative pentose phosphate pathway in TIM. As this pathway is a major source of NADPH for anabolic processes, its enhancement may provide the reducing power required for alkaline phosphatase synthesis, thereby promoting organic P mineralization and contributing to the elevated AP observed at the end of co-growth [39]. FUNGuild analysis further showed enrichment of wood saprotrophs and depletion of mycorrhizal guilds, consistent with increased POD activity, accelerated OM turnover, and a shift from mycorrhizal to saprotrophic dominance. Overall, relay intercropping with maize restructures the rhizosphere microbiome by enriching saprotrophic fungi, suppressing specific bacterial functions, and reorganizing co-occurrence networks, thereby supporting enhanced nutrient buffering and OM accumulation.

### 3.4. Metabolite Profiles and Rhizosphere Signaling

After two consecutive years of relay intercropping, differential metabolites in tobacco rhizosphere soil showed an overall decline, particularly readily utilizable small molecules such as D-camphor and 4-methyl-2-oxopentanoate. Given the concurrent increases in bacterial α-diversity, saprotrophic fungal abundance, and carbon- and nitrogen-acquiring enzyme activities—indicators of higher microbial metabolic activity—this decline likely reflects faster substrate turnover rather than reduced input [40,41,42].

KEGG enrichment analysis showed that differential metabolites were mainly enriched in cyanoamino acid metabolism, cysteine and methionine metabolism, and amino acid biosynthesis pathways (Figure 6c). Activation of cysteine and methionine metabolism, both involving sulfur-containing amino acids, may enhance the antioxidant buffering capacity of rhizosphere soil [43]. These pathways are closely linked to plant stress responses and nitrogen metabolism, suggesting that TIM induces a stress-adaptive metabolic shift in the rhizosphere [44,45].

Among the upregulated metabolites, N-acetyl-L-tryptophan, galactinol, and 3-dehydroshikimic acid had the highest VIP scores (Figure 6b). N-acetyl-L-tryptophan is associated with plant nitrogen metabolism [46], galactinol acts as a protective metabolite under abiotic stress [47], and 3-dehydroshikimic acid indicates enhanced secondary metabolism that may improve disease resistance and environmental adaptability [47]. These metabolites were positively correlated with multiple bacterial and fungal phyla (Figure 7c,d). However, whether they actively shape microbial communities or simply accumulate as byproducts of altered microbial activity cannot be determined from these correlations and requires functional validation.

### 3.5. Integrative Pathways: How Relay Intercropping Promotes Tobacco Growth

PLS-PM showed that the planting system had a significant positive effect on soil metabolites but negligible direct effects on soil nutrients or tobacco growth (Figure 7a). This suggests that TIM promotes plant growth indirectly—mainly through microbe- and metabolite-mediated regulation of nutrient transformation and use—rather than by directly increasing nutrient availability. This is consistent with evidence that root interactions in intercropping systems activate metabolite- and microbe-driven processes that enhance soil enzyme activity and mobilize nutrient pools [48]. Mantel analysis further showed strong correlations between microbial community composition and key soil properties (pH, OM, AN, TN, AP, AK; Figure 7b), indicating that microorganisms act as central mediators linking planting patterns to nutrient dynamics [49], as also reported in maize/cassava intercropping systems [50]. Correlation analysis between dominant microbial phyla and the top 20 differential metabolites identified specific associations. Galactinol was positively associated with multiple bacterial phyla, whereas N-acetyl-L-tryptophan and 3-dehydroshikimic acid were positively associated with several fungal phyla. Galactinol may regulate microbial taxa involved in water and nutrient acquisition, thereby improving plant tolerance to abiotic stress [51]. N-acetyl-L-tryptophan is positively associated with Bacteroides, Faecalibacterium, and basidiomycete spore abundance [52], while 3-dehydroshikimic acid may exert directional regulation on soil fungal communities [53]. These results suggest that microbe–metabolite interactions can alleviate nutrient stress and contribute to a beneficial soil legacy for subsequent crops.

### 3.6. Limitations and Future Directions

This study has several limitations. First, statistical analysis was limited to independent *t*-tests at each time point; repeated-measures analysis was not conducted. Therefore, temporal nutrient patterns (e.g., “decline followed by recovery”) should be interpreted as observed trends rather than statistically confirmed trajectories. Second, although correlations were observed between specific metabolites (e.g., galactinol) and microbial taxa, causality cannot be established. Functional validation—such as exogenous metabolite application or genetic approaches—is needed to confirm regulatory roles. Third, the study was conducted at a single site with sandy loam soil, which limits generalizability across edaphic and climatic conditions. Fourth, the proposed “signaling” role of metabolites remains speculative and requires targeted validation, such as transcriptomic or mutant-based analyses.

## 4. Materials and Methods

### 4.1. Materials

The tobacco variety K326 and the maize variety Tengyi No. 1 were used. Fertilization was applied throughout the experiment according to the schedules provided in Appendix A Table A4.

### 4.2. Experimental Design

Field experiments were conducted during the tobacco-growing seasons (April–October) in 2023 and 2024 in Yong’an Village, Jietou Town, Tengchong City, Yunnan Province (25°18′54″ N, 98°36′54″ E; altitude 1468 m). The soil was classified as sandy loam. Prior to the 2024 season, baseline soil properties were measured as follows: pH 6.5, OM 20 g/kg, alkali-hydrolyzable N (AN) 70 mg·kg^−1^, available phosphorus (AP) 20 mg·kg^−1^, and available potassium (AK) 300 mg·kg^−1^.

A randomized block design with two treatments was used: continuous tobacco monoculture (TM) and tobacco relay intercropped with maize during the harvest period (TIM). Each treatment had three replicates, totaling six plots (100 m^2^ each). The same planting scheme was applied in both years. In TIM, maize was sown on both sides of each tobacco ridge during harvest, with two rows per ridge. Row spacing was 40–45 cm, plant spacing 25–30 cm, and sowing depth 2–5 cm, with 2–3 seeds per hole, resulting in a planting density of ~60,000 plants·ha^−1^.

### 4.3. Sample Collection

Plant height, stem circumference, leaf number, maximum leaf length, and leaf width were measured at 30, 60, and 90 days after transplanting, corresponding to the root elongation, vigorous growth, and maturity stages of tobacco. After tobacco harvest, whole plants were gently uprooted, surface soil was removed, and rhizosphere soil was collected by shaking from the 10–15 cm soil layer. Soil samples were conducted at four time points: before tobacco transplanting, before maize relay intercropping, after the tobacco maize coexistence period following tobacco harvest, and after maize maturity. Each sample weighed at least 200 g and was air-dried in the shade. Additionally, fresh rhizosphere soil collected at tobacco harvest was placed in 10 mL centrifuge tubes, flash frozen in liquid nitrogen for 30 min, and stored at −80 °C for subsequent analyses of soil microbial diversity and metabolites.

Plant samples were collected using a five-point sampling method. At maturity, individual tobacco plants were harvested and separated into roots, stems, lower leaves (foot leaves), middle leaves (waist leaves), and upper leaves (top leaves). Samples were steamed at 105 °C for 30 min, dried at 75 °C to constant weight, ground, and passed through a 0.25 mm sieve for determination of N, P, and K content. The initial cured tobacco leaves were used for chemical quality analysis.

### 4.4. Sample Analysis

#### 4.4.1. Measurement of Soil Agrochemical Properties and Enzyme Activity

Soil samples collected at four different time points were analyzed for their agrochemical properties and enzyme activity. Soil pH was measured using the water extraction method. Soil OM was determined by potassium dichromate titration. AN was analyzed by alkaline hydrolysis diffusion method, AP by the molybdenum-antimony colorimetric method, and AK by ammonium acetate extraction-flame photometry. TN was determined using the semi-micro Kjeldahl method, total P (TP) by perchloric acid digestion, and total K (TK) by sodium hydroxide fusion, following standard procedures [54]. Soil samples were collected from three independent biological replicates per treatment at each time point (n = 3).

Soil enzyme activities, including ACP, POD, SC, UE, CAT, and PPO, were measured using commercial kits (Suzhou Grace Biotechnology Co., Ltd., Suzhou, China) following the manufacturer’s instructions. Assays were conducted in 96-well microplates with appropriate substrates and buffers, incubated at 25–37 °C, and quantified using a microplate reader (SpectraMax M2e, Molecular Devices, San Jose, CA, USA) at 405–578 nm, depending on the enzyme. One unit of activity was defined as 1 μmol product g^−1^ dry soil h^−1^ (ACP, POD, CAT, PPO) or 1 mg product g^−1^ dry soil per 24 h (SC, UE). All samples were analyzed in triplicate.

#### 4.4.2. Measurement of N, P, and K Content in Plants

The contents of N, P, and K in both aboveground and underground plant tissues were determined from dried samples according to the national standard NY/T 2017-2011 (“Determination Methods of Nitrogen, Phosphorus, and Potassium in Plants”). N/P/K accumulation per plant (mg/plant) was calculated as [55]

Accumulation (mg·plant^−1^) = dry weight (g·plant^−1^) × N/P/K content (mg·g^−1^)

#### 4.4.3. Measurement of Chemical Components in Tobacco Leaves

After initial curing, tobacco leaf samples were adjusted to a moisture content of 12 ± 1% at 45 °C, de-stemmed, ground, and sieved through a 0.425 mm (40 mesh) sieve. TN and nicotine contents in the initial cured tobacco leaves were measured using continuous flow analysis according to YC/T 161-2002 and YC/T 160-2002, respectively. The total sugar and reducing sugar contents were determined following YC/T 159-2002. K content was determined by flame photometry (YC/T 173-2003), and chloride content by potentiometric titration (YC/T 162-2011). The potassium-to-chloride, sugar-to-alkali, and nitrogen-to-alkali ratios were calculated. All analyses were performed in triplicate, with relative deviations maintained below 5% [56].

#### 4.4.4. Measurement of Tobacco and Maize Yields

Following the removal of border rows, tobacco plants from each plot were harvested and processed following the national standard GB/T 23219-2008 for grading, hanging, and initial curing. Leaf dry weight was recorded to calculate tobacco yield. After relay intercropping maize reached maturity, yield was measured from a representative area of ≥10 m^2^ per plot, and above-ground fresh weight was recorded. Three randomly selected plants were dried by steaming at 105 °C for 30 min, dried at 75 °C to constant weight, or used to determine moisture content. Aboveground maize yield was then converted to tons per hectare (t·hm^−2^). Economic value was estimated using a local market price of 350 CNY per ton [57].

#### 4.4.5. Analysis of Soil Microbial Communities

At the end of the tobacco harvest, rhizosphere soil was collected from six biological replicates per treatment (n = 6). Total genomic DNA was extracted using the E.Z.N.A.™ Soil DNA Kit (Omega Bio-tek, Norcross, GA, USA), assessed by 0.8% agarose gel electrophoresis, and quantified with a NanoDrop 2000 spectrophotometer (Thermo Fisher Scientific, Waltham, MA, USA). The bacterial 16S rRNA V3–V4 region was amplified using primers 338F (5′-ACTCCTACGGGAGGCAGCA-3′) and 806R (5′-GGACTACHVGGGTWTCTAAT-3′), and the fungal ITS1 region using primers ITS1a (5′-CTTGGTCATTTAGAGGAAGTAA-3′) and ITS1b (5′-GCTGCGTTCTTCATCGATGC-3′). Purified amplicons were sequenced (paired-end) on an Illumina NovaSeq platform (NovaSeq 6000 SP Reagent Kit, 500 cycles, Illumina, Inc., San Diego, CA, USA).

Sequence data were processed in QIIME 2 (v2019.4). Raw reads were demultiplexed, primer- and barcode-trimmed, quality-filtered (Phred ≥ Q20), denoised, and merged using DADA2. Chimeras were removed using the consensus method. Rarefaction curves reached saturation, indicating sufficient sequencing depth. ASVs were classified against the SILVA 138.1 (bacteria) and UNITE 8.0 (fungi) databases at 99% similarity.

#### 4.4.6. Analysis of Soil Metabolites

For soil metabolite analysis, rhizosphere soil samples were collected from six independent biological replicates per treatment (n = 6). Each sample (50 mg) was homogenized in 500 μL of ice-cold 70% (*v*/*v*) methanol/water, incubated on ice for 15 min, and centrifuged at 12,000 rpm for 10 min at 4 °C. The supernatant was collected, and the pellet was re-extracted with 500 μL of ethyl acetate/methanol (1:3, *v*/*v*) under agitation for 5 min, followed by incubation on ice for 15 min and centrifugation under the same conditions. The supernatants were combined, concentrated, dried, and reconstituted in 100 μL of 70% methanol, followed by ultrasonication for 3 min and final centrifugation at 12,000 rpm for 3 min at 4 °C. The resulting supernatant was used for LC–MS/MS analysis.

### 4.5. Data Analysis

#### 4.5.1. Analysis of Soil Agrochemical Properties and Enzyme Activity

Independent samples *t*-tests were used to assess the effects of planting practices on soil agrochemical properties, enzyme activities, and crop growth parameters. Data are presented as means ± standard error (SE). All statistical analyses, including tests for homogeneity of variance and normality, were conducted using SPSS version 22.0, and figures were generated with Origin 2021.

#### 4.5.2. Microbial Community Analysis

Microbial community analyses were conducted in QIIME 2 (version 2019.4). ASV classification was applied to 99% of the Operational Taxonomic Units (OTUs) reference sequences. The α-diversity indices and ß-diversity analyses were calculated based on the ASV level. Gephi version 0.10.1 was used to visualize microbial co-occurrence networks based on strong (|*p*| > 0.6) and significant (*p* < 0.01) correlations.

#### 4.5.3. Soil Metabolite Analysis

Soil metabolomic data were processed and analyzed using the Metware Cloud Analysis Platform (https://cloud.metware.cn, accessed on 14 November 2025) to identify differential metabolites across treatment groups [58]. Differential metabolite screening was conducted using a combination of variance analysis and orthogonal partial least squares discriminant analysis (OPLS-DA), with thresholds of *p* < 0.05 and VIP > 1. The identified differential metabolites were subsequently subjected to KEGG pathway enrichment analysis. Additionally, a PLS-PM analysis was applied to investigate the interactions between rhizosphere microorganisms and metabolites, and to elucidate their integrated effects on soil N, P, and K nutrients.

#### 4.5.4. Correlation Analysis

To examine relationships among planting patterns, soil metabolites, microbial communities, enzyme activities, and soil nutrients, PLS-PM was performed using the plspm package in R (version 4.2.0). The model included five latent variables: planting pattern (TM/TIM, dummy coded), soil metabolites (19 variables), soil microorganisms (40 variables, including 20 bacterial and 20 fungal genera), soil enzyme activities (ACP, POD, SC, UE, CAT, PPO), and soil nutrients (pH, OM, AN, AP, AK, TN, TP, TK). The model was estimated using the path weighting scheme with 500 bootstrap resamples to assess path coefficient significance. Model performance was evaluated using the goodness-of-fit (GoF) index.

A Mantel test was used to assess correlations between soil environmental factors and microbial community composition. Bacterial and fungal distance matrices were calculated using Bray–Curtis dissimilarity based on relative abundances of all ASVs. Environmental variables included pH, OM, AN, TN, AP, and AK. The Mantel test was conducted using the mantel function in the vegan package (version 2.6-4), with Spearman’s rank correlation and 9999 permutations.

Key microbial taxa driving community shifts were identified using Random Forest analysis with the randomForest package (version 4.7-1) in R. Bacterial and fungal phyla with relative abundance >0.1% were used as predictors, and treatment (TM vs. TIM) as the response variable. Taxa importance was ranked using MeanDecreaseAccuracy, and phyla with values >5 were considered key contributors.

Spearman correlation analysis was conducted between key microbial phyla (identified by Random Forest) and the top 20 differential metabolites (ranked by VIP scores from OPLS-DA). Correlation coefficients and *p*-values were calculated using the cor.test function in R. Significant correlations (*p* < 0.05) were visualized as heatmaps using the pheatmap package (version 1.0.12). All statistical analyses were performed using R version 4.2.0 (R Core Team, 2022).

## 5. Conclusions

This two-year field study shows that TIM maintains flue-cured tobacco yield and quality while providing additional economic returns from silage maize (4.86 t·hm^−2^, 1.70 × 10^4^ CNY·hm^−2^, LER = 1.27). TIM reduced N accumulation in tobacco shoots and transiently decreased soil OM and AK, but increased ACP, POD, and UE activities. These changes coincided with higher bacterial α-diversity, enrichment of beneficial genera (e.g., *Candidatus Solibacter*, *Talaromyces*, *Penicillium*), and marked shifts in rhizosphere metabolite profiles. PLS-PM analysis indicates that TIM promotes tobacco growth mainly through microbe- and metabolite-mediated pathways rather than direct changes in soil nutrient availability. Overall, TIM induces a soil legacy effect that shifts the system from direct nutrient competition to microbially mediated nutrient buffering, offering a sustainable model for continuous tobacco production that balances productivity and ecological resilience.

## Figures and Tables

**Table 1:** Agronomic traits of flue-cured tobacco plants.

Period	Treatment	Plant Height (cm)	Stem Circumference (cm)	Number of Leaves	Internode Length (cm)	Maximum Leaf Length (cm)	Maximum Leaf Width (cm)
30 days after transplanting	TM	12.45 ± 1.35	6.27 ± 0.29	9.50 ± 0.73	3.17 ± 0.31	40.85 ± 1.35	23.28 ± 1.59
	TIM	11.55 ± 1.92	6.27 ± 1.82	9.07 ± 0.46	2.81 ± 0.5	39.15 ± 2.39	22.03 ± 1.69
60 days after transplanting	TM	70.98 ± 10.06	8.88 ± 0.43	19.20 ± 1.52	3.01 ± 0.47	61.11 ± 16.12	27.85 ± 3.44
	TIM	79.39 ± 10.39 *	9.24 ± 0.71	19.20 ± 1.61	3.21 ± 0.49	68.69 ± 4.61 **	28.67 ± 3.35
90 days after transplanting	TM	111.5 ± 10.5	10.25 ± 0.52	20.07 ± 2.05	4.3 ± 1.1	81.7 ± 3.76	32.5 ± 4.51
	TIM	114.34 ± 9.17	10.43 ± 0.43	20.13 ± 1.88	4.6 ± 0.93	78.4 ± 5.58	34.13 ± 3.07 *

Note: TM: Continuous tobacco monoculture for two consecutive years; TIM: Continuous tobacco intercropped with maize for two consecutive years. Differences between treatments were analyzed using the independent samples *t*-test. * indicates *p* ≤ 0.05; ** indicates 0.001 ≤ *p* < 0.01.

**Table 2:** Chemical composition of early-cured tobacco leaves (B2F and C3F).

	Nicotine (%)	Chlorine (%)	Reducing Sugar (%)	Total Sugar (%)	Total Nitrogen (%)	Potassium (%)	K/Cl Ratio	Sugar/Nicotine Ratio	Nitrogen/Nicotine Ratio
TM-B	2.30 ± 0.014	0.42 ± 0.01	17.72 ± 0.25	24.17 ± 0.007	2.1 ± 0.02 **	3.01 ± 0.05	7.16 ± 0.37 *	7.97 ± 0.07 **	0.95 ± 0.01 *
TIM-B	2.49 ± 0.08	0.48 ± 0.02 *	17.46 ± 0.5	24.84 ± 0.37	1.94 ± 0.00	3.08 ± 0.08	6.41 ± 0.17	7.15 ± 0.04	0.8 ± 0.03
TM-C	2.24 ± 0.007	0.37 ± 0.003	18.63 ± 0.31	26.31 ± 0.29	1.78 ± 0.14	3.3 ± 0.04	11.97 ± 0.37	8.33 ± 0.18	0.8 ± 0.06
TIM-C	2.46 ± 0.24	0.4 ± 0.004	18.4 ± 0.23	27.29 ± 0.58	1.52 ± 0.03	3.43 ± 0.07	10.68 ± 0.28	7.5 ± 0.63	0.62 ± 0.07

Note: TM and TIM are the same as described in Table 1. Values are means ± SD (n = 3). * indicates *p* ≤ 0.05; ** indicates 0.001 ≤ *p* < 0.01. B denotes B2F grade, C denotes C3F grade.

**Table 3:** Yield and output value statistics of tobacco and subsequent silage maize under different cropping patterns.

Cropping Pattern	Above-Ground Biological Yield (kg·ha^−1^)		Output Value (CNY·ha^−1^)	
	Flue-Cured Tobacco	Subsequent Silage Maize	Flue-Cured Tobacco	Subsequent Silage Maize
TM	1905 ± 68.74	-	66,579.75 ± 2402.42	-
TIM	1900 ± 60.62	48,600 ± 4637	66,405.02 ± 2118.73	17,010 ± 679

Note: TM and TIM are the same as described in Table 1. Values are means ± SD (n = 3). For silage maize, above-ground biomass yield is presented.

**Table 4:** Soil agrochemical properties under different cropping patterns.

Period	Treatment	pH	OM (g·kg^−1^)	AN (mg·100 g^−1^)	AP (mg·kg^−1^)	AK (mg·kg^−1^)	TN (g·kg^−1^)	TP (g·kg^−1^)	TK (g·kg^−1^)
Before transplanting	TM	5.72 ± 0.05	11.76 ± 0.39	17.15 ± 2.47	2.96 ± 0.02	233.4 ± 3.22	2.20 ± 0.06	0.354 ± 0.03	19.08 ± 0.16
	TIM	6.00 ± 0.04 **	11.58 ± 0.18	20.3 ± 0.99 *	3.76 ± 0.21 **	228.6 ± 3.13	2.27 ± 0.12	0.346 ± 0.00	18.91 ± 0.10
Before relay	TM	5.55 ± 0.17	9.59 ± 0.12 *	19.95 ± 1.48	4.70 ± 0.16 **	83.85 ± 0.92	2.31 ± 0.08	0.395 ± 0.03	19.59 ± 0.20
	TIM	6.30 ± 0.16 **	8.64 ± 0.28	19.2 ± 0.99	3.05 ± 0.27	117.55 ± 0.90 **	2.34 ± 0.06	0.382 ± 0.01	19.08 ± 0.90
Co-growth end	TM	6.16 ± 0.05 **	9.31 ± 0.09 **	11.35 ± 1.49	2.41 ± 0.08	233.55 ± 3.89 **	1.28 ± 0.12	0.289 ± 0.3	17.88 ± 0.80
	TIM	5.82 ± 0.04	9.02 ± 0.05	10.85 ± 0.35	2.38 ± 0.09	213.8 ± 1.98	1.23 ± 0.12	0.282 ± 0.5	17.4 ± 2.71
Maize maturity	TM	5.02 ± 0.06	11.94 ± 0.28	21.35 ± 1.48	7.43 ± 0.04	160.4 ± 3.68	2.01 ± 0.26	0.254 ± 0.03	16.32 ± 0.40
	TIM	5.25 ± 0.18	13.79 ± 0.67 **	23.1 ± 1.98	8.98 ± 0.06 **	158.3 ± 6.93	1.95 ± 0.18	0.247 ± 0.01	15.94 ± 0.80

Note: OM, organic matter; AN, alkali-hydrolyzable nitrogen; AP, available phosphorus; AK, available potassium; TN, total nitrogen; TP, total phosphorus; TK, total potassium. TM and TIM are the same as described in Table 1. Values are means ± SD (n = 3). * indicates *p* ≤ 0.05; ** indicates 0.001 ≤ *p* < 0.01.

## Data Availability

The datasets generated during and analyzed during the current study are not publicly available due to ongoing research or data privacy agreements but are available from the corresponding author upon reasonable request.

## Appendix A

**Table A1:** Topological parameters of co-occurrence networks under different patterns.

Index	TM	TIM
Modularity (MD)	0.519	0.566
Average clustering coefficient	0.564	0.492
Average path length	2.596	2.966
Grath density	0.123	0.092
Average degree (AD)	11.667	8.653
Nodes	96	95
Edges	560	411
Positive correlation edges	389	276
Negative correlation edges	171	135

Note: TM, continuous tobacco monoculture for two consecutive years; TIM, continuous tobacco inter-cropped with maize for two consecutive years.

**Table A2:** Abundance table of the top 15 predicted bacterial functions based on the PICRUSt2 pathway.

#Function	TIM	TM	Description
PWY-3781	564,131.6	552,637.5	Aerobic respiration I (cytochrome c)
PWY-7111	314,947.3	320,114.6	Pyruvate fermentation to isobutanol (engineered)
PWY-5101	306,800.3	310,548.8	L-isoleucine biosynthesis II
VALSYN-PWY	296,015.6	300,066.8	L-valine biosynthesis
ILEUSYN-PWY	296,015.6	300,066.8	L-isoleucine biosynthesis I (from threonine)
PWY-5973	257,854.2	266,598.6	cis-vaccenate biosynthesis
PWY-7663	251,668.5	261,167.4	Gondoate biosynthesis (anaerobic)
BRANCHED-CHAIN-AA-SYN-PWY	256,332.1	256,116.2	Super-pathway of branched amino acid biosynthesis
PWY-7094	240,024.4	246,812.6	Fatty acid salvage
FASYN-ELONG-PWY	238,339.7	244,914.6	Fatty acid elongation—saturated
FAO-PWY	231,638.4	240,864.5	Fatty acid &beta;-oxidation I
TCA	237,272	236,400.8	TCA cycle I (prokaryotic)
PWY-6969	236,873.7	233,726.8	TCA cycle V (2-oxoglutarate:ferredoxin oxidoreductase)
NONOXIPENT-PWY	234,195.6	223,295.8	Pentose phosphate pathway (non-oxidative branch)
PWY0-1319	232,768.6	225,235.8	CDP-diacylglycerol biosynthesis II

Note: TM, continuous tobacco monoculture for two consecutive years; TIM, continuous tobacco inter-cropped with maize for two consecutive years.

**Table A3:** Abundance table of the top 15 predicted fungal functions at the guild level based on the FUNGuild database.

Feature	TM	TIM	Description
Unassigned	350,570	333,762	Unassigned
Undefined_Saprotroph-Wood_Saprotroph	144,357	88,212	Undefined_Saprotroph-Wood_Saprotroph
Undefined_Saprotroph	73,020	85,392	Undefined_Saprotroph
Animal_Pathogen-Dung_Saprotroph-Endophyte-Plant_Saprotroph-Soil_Saprotroph-Wood_Saprotroph	3498	52,091	Animal_Pathogen-Dung_Saprotroph-Endophyte-Plant_Saprotroph-Soil_Saprotroph-Wood_Saprotroph
Fungal_Parasite-Undefined_Saprotroph	12,004	37,631	Fungal_Parasite-Undefined_Saprotroph
Plant_Pathogen-Soil_Saprotroph-Wood_Saprotroph	21,429	15,756	Plant_Pathogen-Soil_Saprotroph-Wood_Saprotroph
Dung_Saprotroph	11,866	9198	Dung_Saprotroph
Plant_Pathogen	6703	11,794	Plant_Pathogen
Wood_Saprotroph	5013	623	Wood_Saprotroph
Dung_Saprotroph-Undefined_Saprotroph-Wood_Saprotroph	4345	210	Dung_Saprotroph-Undefined_Saprotroph-Wood_Saprotroph
Dung_Saprotroph-Wood_Saprotroph	2288	3548	Dung_Saprotroph-Wood_Saprotroph
Ectomycorrhizal	3472	3479	Ectomycorrhizal
Endophyte-Plant_Pathogen-Wood_Saprotroph	3064	222	Endophyte-Plant_Pathogen-Wood_Saprotroph
Clavicipitaceous_Endophyte-Plant_Pathogen	1462	2622	Clavicipitaceous_Endophyte-Plant_Pathogen
Ericoid_Mycorrhizal	2420	2096	Ericoid_Mycorrhizal

Note: TM, continuous tobacco monoculture for two consecutive years; TIM, continuous tobacco inter-cropped with maize for two consecutive years.

**Table A4:** Fertilization rates for flue-cured tobacco under different cropping patterns.

N:P:K Ratio	Fertilization Rate (kg·hm^−2^)	Application Date
Compound Fertilizer (10:16:26)	300	16 April
Water-Soluble Fertilizer of Major Elements (15:16:25)	75	16 May
Water-Soluble Fertilizer of Major Elements (15:16:25)	150	25 June
Compound Fertilizer (10:16:26)	375	28 August
